# Decision-Making of Older Patients in Context of the Doctor-Patient Relationship: A Typology Ranging from “Self-Determined” to “Doctor-Trusting” Patients

**DOI:** 10.1155/2013/478498

**Published:** 2013-04-18

**Authors:** Jennifer Wrede-Sach, Isabel Voigt, Heike Diederichs-Egidi, Eva Hummers-Pradier, Marie-Luise Dierks, Ulrike Junius-Walker

**Affiliations:** ^1^Hannover Medical School, Institute for General Practice, Carl-Neuberg Street 1, 30625 Hannover, Germany; ^2^University Medical Center Hamburg-Eppendorf, Department of Primary Medical Care, Martinistr. 52, 20246 Hamburg, Germany; ^3^University Medical Centre Göttingen, Department of General Practice, Humboldtallee 38, 37073 Göttingen, Germany; ^4^Hannover Medical School, Institute for Epidemiology, Public Medicine and Healthcare Systems Research, Carl-Neuberg Street 1, 30625 Hannover, Germany

## Abstract

*Background*. This qualitative study aims to gain insight into the perceptions and experiences of older patients with regard to sharing health care decisions with their general practitioners. *Patients and Methods*. Thirty-four general practice patients (≥70 years) were asked about their preferences and experiences concerning shared decision making with their doctors using qualitative semistructured interviews. All interviews were analysed according to principles of content analysis. The resulting categories were then arranged into a classification grid to develop a typology of preferences for participating in decision-making processes. *Results*. Older patients generally preferred to make decisions concerning everyday life rather than medical decisions, which they preferred to leave to their doctors. We characterised eight different patient types based on four interdependent positions (self-determination, adherence, information seeking, and trust). Experiences of a good doctor-patient relationship were associated with trust, reliance on the doctor for information and decision making, and adherence. *Conclusion*. Owing to the varied patient decision-making types, it is not easy for doctors to anticipate the desired level of patient involvement. However, the decision matter and the self-determination of patients provide good starting points in preparing the ground for shared decision making. A good relationship with the doctor facilitates satisfying decision-making experiences.

## 1. Introduction

One challenge in primary care in Germany is the treatment of older patients who consult their general practitioners (GPs) with multiple health and associated everyday problems [1–3]. Shared decision making (SDM) is a particular communication strategy in the doctor-patient dialogue with medical decisions being taken jointly by the doctor and the patient. This concept involves the doctor ascertaining the needs of the patient, providing the patient with information and explaining the various preferred methods of treatment. In this way the personal values and preferences of the patient can be taken into account [4–6]. The approach is seen as a key element in patient-centered care and the favoured model of decision making [7–9]. It has been tailored for use in general practice [10]. Patient centeredness contributes to better patient knowledge and more realistic patient expectations about the course of the disease, more active patient participation in the treatment process, and fewer decisional conflicts [11]. For the doctor, actively inquiring after the patient perspective during the consultation has lead to a decrease in questionable prescribing of antibiotics [12]. Nevertheless, patient-centered care has been studied more often in doctors caring for healthier patients [13]. Numerous studies demonstrate that most patients, if they were asked, would like to play an active role in health care decisions [14]. However, it has also been shown that patient behaviour in actual decision making situations does, in fact, diverge from the “ideal” formulated by the patient [15]. This is especially the case for older people, who tend to have a lower preference to participate in such decisions than younger people [16]. According to a European study [17], it is not so much actively participating in the decision making that is important for older patients but rather the desire for a trusting relationship with the doctor, a patient-centred approach (e.g., the interest of the doctor, a trustworthy and supportive manner), and receiving information. The study emphasises that the desire for involvement is heterogeneous in the group of over 70 years old and that this can change over time and during the course of illness. This means that, in practice, it is necessary to find a flexible approach in which the doctor caters to the individual needs of older patients.

The aim of this study is to develop a theoretical model of the perceptions and experiences of older patients with regard to sharing health care decisions with their GPs in context of their relationship. We therefore conducted and analyzed semistructured interviews with older general practice patients that led to a classification grid in which the patients' preferred level of participation and their reported actual behaviour in decision-making situations are presented.

## 2. Materials and Methods

The study was conducted between September 2008 and January 2009. The qualitative data was obtained in the first part of the research project “PrefCheck: Preferences in treatment planning for older patients” (German Trial Register: DRKS 00000792) [18]. The study was approved by the ethics committee of the Hannover Medical School (no. 5069). 

### 2.1. Recruitment of the Study Participants

Nine general practices in the area of Hannover (City of Hannover/southern regions) participated in the study. While four GPs were recruited through professional contacts of the authors, the others were respondents to cover letters with information leaflets sent to a random selection of 30 GPs (50% female) with their own practice. Each practice recruited four patients. Statistical sampling was used to select the participants, who were women and men in the age groups <80 years old or ≥80 years old. Each patient who visited the practice from 10 am during the recruitment week and met the inclusion criteria (aged at least 70 years) was approached consecutively. Exclusion criteria were history of severe cognitive impairment, legal incapacity, inadequate knowledge of German or inadequate language competence, profound hearing loss, currently participating in another clinical study, not reachable by telephone, and categorised as nursing care level II or III (no independent living possible). Participating patients were informed about the aims of the study, signed a written permission, and agreed to provide sociodemographic data.

### 2.2. Data Collection

The data collection took place in autumn 2008. The patients underwent a geriatric assessment (STEP) [19] in the general practitioner's surgery. The geriatric assessment is a multidimensional, multidisciplinary diagnostic instrument designed to collect data on the health and everyday problems of older patients. Two days afterwards, the participants were asked from a member of the research staff on the basis of their assessment results about their view on the health problems (not part of this analysis) and about participation preferences and any experiences they had related to these. This took the form of semistructured interviews in the homes of the participants.

### 2.3. Semistructured Interviews

A structured guide formed the basis of the qualitative interviews and included questions on the patients' preferred level of participation and the patients' actual experiences in this respect in the context of their own medical history.

#### 2.3.1. Semistructured Interview Part 1: Hypothetical Decision Making Situation

The first part of the interviews entailed the presentation of general fictitious decision making scenarios (see Box 1), which were developed from experience prior to this project in interviews with very old people [20]. The scenarios were designed to take in different areas of health care decision making: purely medical decisions and also health-related decisions in everyday life. The presentation of the scenario always led to the question: “Who is to decide what should be done?” This initiated a discussion with the interviewees on their reasons and opinions concerning this. To facilitate a better understanding of the decision making scenarios, which were kept rather general, the personal assessment results provided good illustrations in the course of the interview.

#### 2.3.2. Semistructured Interview Part 2: Patient Behaviour in Personally Experienced Decision Making Situations

In the next part of the interview, the patients were asked to give an account of their experiences in participating in decision making. The key question was: “Has there ever been a difference of opinion between you and your doctor as regards the choice of medical treatment? If so, what happened? Please tell me about it.”

The interviews were recorded on a minidisc player. 

### 2.4. Analysis of the Interviews

The recorded interviews were transcribed (simple transcription) and analysed using the analysis programme Atlas.ti. Samples of the entire interviews were checked against the transcripts for accuracy. 

A two-stage procedure that integrated a qualitative content analysis [21] and a type construction scheme [22] was chosen for the analysis, which was independently carried out by two research associates together with a four-member research team. The results and interpretations were compared and discussed before final conclusions were drawn.

#### 2.4.1. Analysis Step 1: Content Analysis

Firstly, a qualitative content analysis was conducted in order to reduce and compress the volume of data within the context of the research question. For this purpose, all the sections of the interviews referring to the “fictitious decision-making scenarios” and to other reported experiences were extracted and used for an analysis covering all the patients. This entailed identifying the particular categories relating to “decision-making”, “autonomy and self-determination,” and “dealing with differences in opinion” and summarising these in a table. An overview of the categories, subcategories, and codes can be found in Table 1.

#### 2.4.2. Analysis Step 2: Type Construction

A “multistage model of empirically grounded type construction” [22] was used to process the reorganised material. This approach attempts to construct “ideal types.” For this purpose, first, relevant dimensions and manifestations for comparison are developed. The cases are then grouped and analysed for their empirical regularities in order, finally, to obtain “typical cases” [23]. These “ideal types” represent a notional, one-dimensional enhancement of specific typical elements that, although they are gained from real contexts, cannot be found in real people to this extent [24, 25].

To develop a typology from the patient interviews, the participation preferences expressed by each interviewee were set in relation to his reported (i.e., actual) behaviour in the dealings with doctors. These two dimensions (“decision-making preference,” “adherence to doctor's advice”) resulted from the information on decision-making preferences in the fictitious scenarios as well as from the situations described by the patients in which there was a difference in opinion between the patient and the doctor. The next two dimensions, “trust in doctors,” and “desire for information,” were identified as the central themes in the analysis of the material across all patients and were used in the typology classification because of the frequency of their occurrence in all the interviews (see Table 2).

In the next step, the interview of each patient was reexamined in the light of the grid that had been developed. Each relevant section of the interview material, which had earlier been sorted into tabular form, was matched to the appropriate manifestation of the four dimensions. An individual combination of the various manifestations of the dimensions was thus obtained for each interviewed patient.

Clusters of interviewed patients could then be identified, on the basis of which (ideal) patient types were characterised in a final step. The two dimensions “decision-making preference” and “adherence to doctor's advice” were chosen as the main categories for this in a coordinate system to represent the different types on the *x*-axis and the *y*-axis. These two dimensions were defined as the main group categories since they allowed the elderly patients' preference for participation in decision making to be related to their reported actual behaviour. The two other dimensions, “trust in doctors” and “desire for information,” served the purpose of describing the respective patient types more precisely.

Finally, all patient interviews were revisited to extract information about experienced doctor-patient relationships to explore connections with the respective decision-making type. 

## 3. Results

In the nine general practices, 48 patients were approached, 34 (71%) of whom were included in the study and interviewed. The average duration of the interviews was 27 minutes (range: 11–50 minutes).  Table 3 summarises the sociodemographic details of the interviewed patients. The level of education was classified according to the recommendations of the Robert Koch Institute [26]. 

The first part of the following presentation of the results refers to the patients' preferred participation in decision making and is arranged according to the various fictitious decision-making scenarios of the semistructured interviews; the second part introduces the patient types that were developed.

### 3.1. Results Analysis Step 1: Content Analysis of the Semistructured Interviews

#### 3.1.1. Different Options for Treating an Illness

In the case of different treatment options for an illness, the interviewees did not form a homogeneous group. Most patients assumed that the doctor would make the decisions in the fictitious decision-making scenarios: “… *if the decisions are important, then he makes them alone anyway* [the doctor]” (female, 73 years old). Few interviewees found it important to decide for themselves in this situation: “*I don't think anyone would decide this, I have to decide it for myself*” (female, 79 years old). At the same time it was also clear that it was necessary to have been informed calmly, comprehensively, and in an understandable way by the doctor before a decision: “*She* [the doctor] *should give me a good explanation of what the alternatives are*” (male, 80 years old). Regardless of the decision-making preference, a relationship of trust—often longstanding—with the doctor was important for many of the interviewed patients: “…* I have always trusted the doctor*” (female, 85 years old). 

#### 3.1.2. Further Tests (e.g., an X-Ray) Are to Be Made

If a further examination was needed (e.g., an X-ray examination), the patients were unanimous in saying that this must be decided by the doctor. It was noticeable that most of the elderly patients could not imagine having a say in this matter. One patient (female, 87 years old) commented: “*(…) Who else should do it? Well, I can't make the decision, I don't even know if it's needed that urgently, do I? And if the doctor says we have to check that or that has to be observed, we need to X-ray, well then I have an X-ray*.”

The reason given for the doctor having to make this decision was the status of the doctor as “the expert” and the patient as “the layman,” who lacked the necessary expertise to make such decisions. It is precisely because the doctor should make the decisions that the patient consults him.

#### 3.1.3. New Medication

Also in the case of taking new medication, it is the doctor, as the expert, who should make the decisions. A patient (female, 87 years old) made such a comment: “*No, in that sense I'm a layperson. So I have no idea what is good for me, and there are really so many medicines on the market, how should I decide what's right for me? That's what I really have to leave up to the doctor/so I really do need to have enough trust to leave it up to the doctor*.” Some patients suggested that, if there were problems with the intake of medication, the doctor would definitely be consulted again in order to receive further information: “*Yes, of course, sure I would grab the patient information leaflet and look up what it is. And then if I had any difficulties or questions, I (…) say: this and that and that, explain it to me once again*” (male, 81 years old).

#### 3.1.4. Installation of Grab Rails at Home

A different picture arises when the fictitious decision concerns modifications to one's own home (e.g., mounting of grab rails). In the world of everyday matters affecting the patient, the interviewed patients almost exclusively wanted to make the decision alone: “*Yes, well I would have to decide that myself*. *(…) I would notice, whether with grab rails or without I … so I decided that myself, because it just wasn't possible anymore …*” (female, 87 years old). 

A clear reason for this was almost never provided. Most of the participants considered the rearrangement of their living environment to be a topic that did not fall within the scope of primary care.

#### 3.1.5. Utilisation of Nursing Care Services

Almost all patients wanted to make up their own mind in this decision-making scenario as well, even if they were forced into this decision by complex constraints. On the topic of using nursing care services, patients frequently expressed their reluctance to move into an old people's home: “*So go into an old people's home now, I'm not at all keen on that (…)*” (male, 80 years old). This manifested itself specifically in the fear of not being able to cope with everyday life alone: “*Yes, I'd like to stay in my flat as long as possible*” (male, 84 years old). At several points, the interviewees mentioned their concerns about losing their independence and thus not being able to make decisions themselves. One patient (female, 79 years old): “*I (…) always need help and it would be very important, very, very important for me that I could manage to live without any help*.” It was striking that, for a number of participants, the word “nursing care” triggered an association with the old people's home or the loss of autonomy and generated fears.

#### 3.1.6. Fictitious Decision-Making Scenarios and Reported Experience

When considering all of the interviews together, differences could be found between the responses to the fictitious situations and the accounts of decision-making situations experienced in the patients' own medical history. This will be illustrated with one case example.

In the fictitious decision-making scenarios (different treatment options, taking an X-ray, intake of new medication, and utilisation of nursing care services), it was the opinion of the patient (female, 81 years old) that the doctor should make the decision: “*I'd like to say, also the doctor. This is because he must prescribe it and he suggests it. (…) No, I don't understand much about it anyway*.” Later, in the course of the interview, the same patient reported on a real experience in which she acted against the doctor's advice: “‘*You can't just stop taking it and keep it!' I say to him: ‘Why not? If I don't have any pain, I don't need any painkiller either!'*” Thus the patient had not verbalized her resistance and confronted the doctor at the time he prescribed the medication but rather decided not to comply.

 In summary, on being asked about their behaviour in the fictitious “Who is to decide what should be done?” situations, participants more frequently stated that the doctor should make the health care decisions; in account of comparable, specifically experienced situations, however, they describe the opposite. Thus, in some cases, older patients act more self-determinedly than they describe in hypothetical situations or than they would imagine themselves to. 

Therefore, in the next step, the two aspects “ideal” and “concrete behaviour” will be used for a more precise characterisation of patient types.

### 3.2. Results Analysis Step 2: Patient Typology on Decision Making

It became apparent that different patient types evolved not only because of the personal attitude towards participation but also in response to the relationship experienced in consultations and particularly in decision-making situations. To illustrate the two derivations, two extreme examples are given.

1. Personal attitude: “I make the decision—as a basic principle. Any doctor can recommend something and I listen to it and/but the last decision is on my side.” (Patient 33).
2. Experience: “I have trusted my doctor. We had a good relationship. He once cleared my heel spur overnight. I phoned him (and I was allowed to phone him anytime) to say: “Doctor, I just wanted to thank you ….” And this with a homeopathic remedy. First I had not believed that it worked.” (Patient 43).

Trust evolved as a response to a good doctor-patient relationship, and it helped patients to be more relaxed with decisions and led to better adherence. The patients often pointed out how doctors contribute to a good relationship: provide time, listen, pay attention and be open (patients 7 and 12), explain and give information (patients 14, 42, and 45), be truthful (patients 11, 14, and 32), be reassuring (patient 46), be a long-standing companion and share experiences (patients 13, 27, 28, and 45), know the patient through and through (patients 27, 28, 32, and 45), and be at eye level (Patient 33).

**Figure 1:**
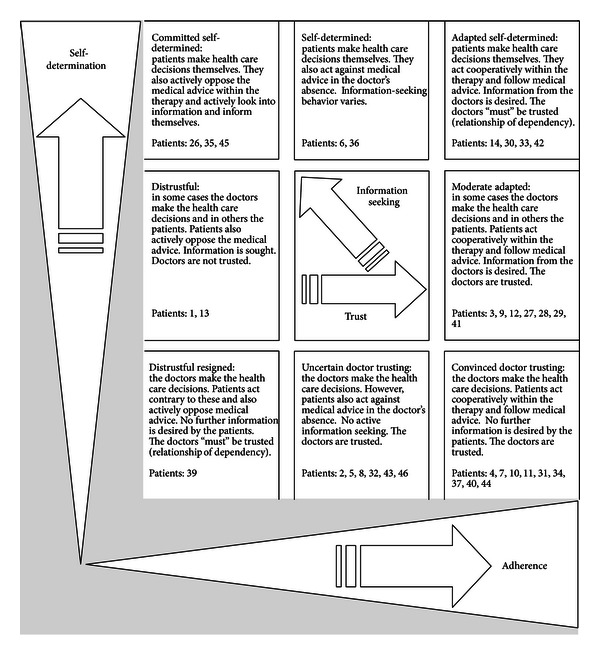
Overview of the patient types (ideal types), classified by the two main categories (self-determination and adherence) and the two additional categories (trust and information-seeking behaviour).

Hence it needs to be taken into account that the patient typology on decision making is often based on the two aspects, which are patients' personal attitudes and the context, in particular the experienced relationship with the doctor. On the basis of the different dimensions (self-determined decision making, adherence, trust, desire for information), eight different ideal types could be found with different degrees of preferences in the decision-making process: preference for patient led decisions (three types), for doctor led decisions (three types), and for decisions with variable participation (two types), (see Figure 1). 

#### 3.2.1. Patients Make the Decisions

Three ideal patient types can be defined for patients who would like to make the health care decisions themselves (Figure 1).

*Committed Self-Determined*. Patient 26 reported that rheumatism had been misdiagnosed by a specialist. For 14 years he took compromising medications. *“(…) have learnt a great deal. That I am very self-confident with the doctor and don't believe everything that they tell me. Lots of questions. The advantages and disadvantages. (…) would rather inform myself on the internet.”* Patient 26 (male, 78 years old).

The “*committed self-determined*” are the first type: those who assert their own decisions even against the doctor. At the same time, they actively inform themselves and discuss the matter critically with the doctors. Distrust prevails.

*Self-Determined*. “*Mrs F. *[the doctor]* described a tablet for blood sugar (…). But then [I] have had dizziness, [I] measured the blood pressure (…) and it was low. I tell myself, I do not need to take the tablet against sugar. Then I stopped taking the tablet by myself*.” Patient 6 (female, 79 years old).

The “*self-determined*” make up the second group in the classification grid. These patients make the decisions themselves and also act against the doctor's advice in his absence.

*Adapted Self-Determined*. Patient 42 talked about her change of antihypertensive medication and admitted that she did not understand the doctor's explanations. “*I only agreed with him because I think he is the one who should know (…).*” Patient 42 (female, 77 years old).

The “*adapted self-determined*” represent the third type in this group. Although these patients would also like to make their own decisions, they act cooperatively within the therapy most of the time and follow the doctor's advice. Patients in this group tend to feel that they are in a relationship of dependency on the doctor.

#### 3.2.2. Patients and Doctors Make the Decisions

Patients who, in part, would like to make the health care decisions themselves and, in part, want a decision from the doctor are divided into the two subgroups “distrustful” and “moderate adapted.”

*Distrustful*. Patient 13 recalled an incidence with a bleeding nipple. After some tests her gynaecologist wanted to wait. She went for a second opinion and later returned postoperatively. When asked about shared decision making, she replied: “*My opinion should also be valued. These days they can't do whatever they want with me anymore.*” Patient 13 (female, 72 years old).

The “*distrustful*” type is characterised by the fact that there is no consistent decision-making preference. The patient also actively opposes the medical advice and does not place trust in the doctor.

*Moderate Adapted*. “*What's important is, when the doctor says that needs to be done, then it is done (…). It is me and of course the doctor, who decide.*” Patient 9 (female, 73 years old).

Quite a few patients can be assigned to the “*moderate adapted.*” Decisions are taken partly by the doctor and partly by the patients in this group, too. The patients invariably act cooperatively and follow the doctor's advice in the course of the therapy. They want information from the doctor, but there is no active discussion or questioning of this information on the part of the patient. The patients place their trust in the doctor.

#### 3.2.3. The Doctors Make the Decisions

The following three ideal types of patients are of the opinion that the doctors should make the decisions.

*Distrustful Resigned*. Patient 39 reflected on his information-seeking behaviour in connection with a prostate operation: “*This is my deficiency that I do not want to know (…) what should I speak to them about, they won't do it differently anyway.*” Patient 39 (male, 73 years old).

According to the “*distrustful resigned*”, it is the doctor who makes the health care decisions. The doctors “must” be trusted (relationship of dependency) and at the same time they actively oppose the doctor's advice. No further information is desired by the patients.

*Uncertain Doctor Trusting*. “*(…) and then I try it, whether I tolerate it or not. And if I don't tolerate it, I simply stop taking it.*” Patient 43 (female, 85 years old).

Much the same is true of the group “*uncertain doctor-trusting.*” The doctors are trusted. Although instructions from the doctor are not actively opposed, the patients describe situations in which, in his absence, they acted against the doctor's advice.

It was possible to gather the various reasons for patients acting against the doctor's advice in his absence from the interview material. Among these were that the doctor did not consider patients' objections, patients wanted to have more self-determination but could not assert themselves against the doctor, or patients did not dare to ask follow-up questions, for example, concerning problems with the therapy or concerns about possible side effects of the medication.

*Convinced Doctor Trusting*. “Dr. M. has diagnosed it and we put it into practice that way. (…) I would rely on the doctor on what he says take this or that [tablet].” Patient 7 (male, 80 years old).

Most of the patients interviewed belong to the groups in which the doctor alone should make the medical decisions. Complete trust is placed in him. Moreover, no further information is desired by the patient and the doctor's instructions are followed. 

## 4. Discussion

In this study, patients were interviewed about their participation preferences in fictitious decision-making scenarios and their experiences in actual decision-making situations with their doctors. Overall, the results reinforce that, when asked about their preference for involvement, the group of older patients differs (cf. 17). However, the results suggest that in tendency older patients are more likely to remain passive and let the doctor make the decisions. This tallies with an American study in which a typology is also developed (Flynn et al., 2006 [27]) and which comes to the conclusion that with increasing age patients tend to leave medical decisions to the doctor. There the authors distinguish between autonomous patients and patients who would rather delegate the decision-making to the doctor. A similar classification into passive and active patient behaviour in decision-making situations is found in Scheibler [28]. Flynn's study group points out that the preference for involvement changes over time. Similarly, in our study it was also noticeable that, for example, the current attitudes of the patient group “(resigned) distrustful”, in particular, have been strongly influenced by previous negative experience. On the other hand, experiences of a good doctor-patient relationship facilitated a convinced doctor-trusting attitude.

Our results clearly demonstrate that the desire for involvement in the decision-making process depends on the matter to be decided. This is also described by Whitney et al. [29]. In their “typology of shared decision making,” the research team classifies decision-making situations according to the criteria risk (between low and high risk) and certainty (between one realistic choice and between more alternatives). Our older patients show a lower involvement in medical decisions as compared to health-related everyday life decisions, which they tend to not share. Older patients' desire for self-determination in their own everyday lives is great. A decision by the doctor that affects issues at home is connected to the risk of losing autonomy (cf. 30). It also seems unexpected since, according to patient opinion, the responsibility for such a decision lies on the patient. This is not the case with disease-related decisions. Our results reinforce those of an American study [30] which show that a forced involvement of the patient in disease-related decisions by the doctor can even lead to resistance on the part of the patient. 

Our patient typology targets older patients and further differentiates existing typologies that represent all age groups. Beyond the distinction between active and passive patient types, our typology includes elements, such as adherence, trust, and information-seeking behaviour. The two latter elements (“trust in medicine” and “desire for information”) have also been identified by Marstedt et al. [31] to describe four patient types (indifferent, doctor-trusting, doctor critical and cotherapist). 

Using the developed classification grid in our study, we have found eight different patient types in relation to decision-making preferences and actual health care participation of older patients. This typology shows heterogeneity of preferences and behaviour not only with regard to involvement in health decisions but also with respect to the actual agreement or opposition to doctors' recommendations and patient adherence. The elements “desire for information” and “degree of trust” are found to relate to patient's practiced autonomy. It seems that little trust in doctors motivates patients to seek information outside the doctor-patient relationship and encourages opposition to medical advice. A doctor-trusting relationship seems to meet patients' information needs already and enhances adherence. The interviewed patients' understanding of the concept of “trust” does, however, vary. Some of the patients state that they see themselves in a relationship of dependency on the doctor and have no alternative but to trust him, whereas others will happily and willingly trust their doctors (cf. 33).

*Limitations*. Patients were chosen from nine practices, each recruiting four. Therefore results should not readily be generalized to practices in other regions. However, this study serves as a pilot into a differentiated (ideal-) patient typology. Statements concerning the frequency of these patient types in the elderly population cannot be made on the basis of the results of our study. This is an area requiring further quantitative research. 

Time as an important factor in the often longitudinal decision-making processes has not been explored sufficiently here. Further research is needed to determine its influence on decision-making types. We are also aware that “patient centeredness” has no universally accepted definition, and we deal with a lack of conceptual clarity for this term [32, 33].

## 5. Conclusions

It is not always obvious to doctors which “type” of patient they have in front of them at any particular time. However, we identified elements essential for decision-making process with older patients. Firstly, self-determined patients tend to actively make health-related decisions. We found that a motive can be either distrust in the context of bad experiences with doctors' decisions or a personal attitude independent of the specific doctor-patient relationship. Secondly, the less self-determined patients tend to rely on doctors making health-related decisions. Whereas a trusting doctor-patient relationship satisfies patients' wishes for information and leads to adherence, nonadherence, and opposition accompany distrust. 

Trust evolves from experiences of a good doctor-patient relationship. Our patients determined a relationship as positive, if they received personal attention and adequate information, truthfulness, empathy, and if they were able to look back at difficult situations that have been mastered together.

Giving adequate information is of particular value not only for the doctor-patient relationship but also for decision-making situations [34, 35]. In practice information needs to be offered in such a way that patients can properly assess it and weigh it up. Social and structural factors need to be taken into account: for instance, the social background of the patient, the amount of time available for the consultation, access to further information, and, finally, ancillary services (cf. 36). 

Three barriers relevant to shared decision making were observed in this group of older patients: one is the patients' dependent trust in authority that inhibits to speak out if they are of a different opinion to the doctor. The second one is the assumption that for some older patients there is only a right decision and a wrong decision—weighing up and discussing different options represent a pattern of behaviour with which these older patients seem to be unfamiliar. Finally, pending decisions in everyday life are not easily raised by the patient for fear of losing autonomy.

Our typology may facilitate the doctor to share decisions with older patients in ensuring that information is sufficiently provided and emotional support is established within a trusting relationship to the degree depending on the patient's preferences. It is apparent that, for one or more of the reasons mentioned previously, older patients sometimes neither desire nor regard shared decision making as possible. In this case, the doctor should be prepared to assess barriers, to reiterate information regarding alternatives, and to respect patient decisions even if the alternatives seem more promising. This open-minded process enhances a trusting relationship, a requirement for such patient-centered care (see [36, 37]).

Successful communication continues to be the key to the realisation of a good doctor-patient relationship and to the design of medical care in line with patient needs [8, 14, 38]. This requires extensive and practically oriented training for doctors. On the other hand, it is necessary to encourage (older) patients to openly express their needs and wishes themselves and to support this.

## Figures and Tables

**Box 1:**
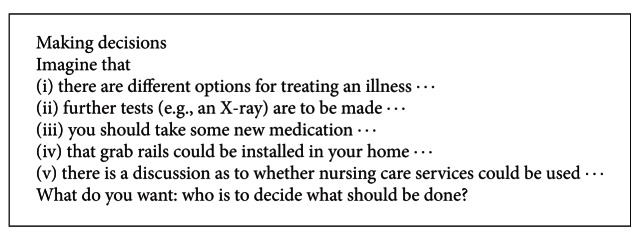
Fictitious decision-making scenarios.

**Table 1:** Categories, subcategories, and codes.

Theme	Attitudes, preferences, and experiences concerning shared decision making							
Categories	Fictitious decision-making scenarios					Reported actual behaviour in decision-making situations		
Subcategories	Different possibilities for treating an illness	Further tests (e.g., an X-ray) are to be made	Take some new medication	Grab rails could be installed	Nursing care services could be used	Decision making	Autonomy and self-determination	Dealing with differences in opinion
Main codes	Decision: patient doctor patient + doctor	Decision: patient doctor patient + doctor	Decision: patient doctor patient + doctor	Decision: patient doctor patient + doctor	Decision: patient doctor patient + doctor	Preference, relationship	Self-determined behaviour, desire for information, relationship	Differences, trust in doctors, adherence, relationship

**Table 2:** Criteria for the construction of patient types.

Dimensions	Manifestation
*Decision-making preference* According to the patient, who makes the health care decisions?	The patient makes the decisions
	The decisions are made partly by the doctor, partly by the patient
	The doctor makes the decisions
*Adherence to doctor's advice* Does the patient cooperate in the therapy and follow the doctor's advice?	Adherence
	Passively against the doctor (does not, e.g., take his medication and does not inform the doctor)
	Actively against the doctor (e.g., leaves the hospital at his own request)
*Trust in doctors* Does the patient have trust in the doctor?	Trust
	Dependence
	No trust
*Desire for information* To what extent does the patient want to receive further information?	Active exchange (discussion/several sources)
	One-way exchange of information from doctor
	No desire for information

**Table 3:** Age, gender, and level of education of the study participants.

Age	Education*	Gender		Total
		female (*n* = 17)	Male (*n* = 17)	
<80 years	Low	3	0	3
	Medium	6	7	13
	High	0	2	2

	Total	9 (50%)	9 (50%)	18 (53%)

≥80 years	Low	3	1	4
	Medium	5	3	8
	High	0	4	4

	Total	8 (50%)	8 (50%)	16 (47%)

*“Education” is measured by the highest school-leaving qualification. Low: a lower secondary education at most, medium: secondary school certificate or completed polytechnic school, and high: qualification for admission to a university or a university for applied sciences.

## References

[1] Fortin M, Lapointe L, Hudon C, Vanasse A, Ntetu AL, Maltais D (2004). Multimorbidity and quality of life in primary care: a systematic review. *Health and Quality of Life Outcomes*.

[2] Tinetti ME, Fried T (2004). The end of the disease era. *American Journal of Medicine*.

[3] Wiesner G, Bittner E (2005). On the incidence and prevalence of polypathia in Germany. *Arbeitsmedizin Sozialmedizin Umweltmedizin*.

[4] Elwyn G, Edwards A, Rhydderch M, Härter M, Loh A, Spies C (2005). Shared decision making: das Konzept und seine Anwendung in der klinischen Praxis. *Gemeinsam Entscheiden—Erfolgreich Behandeln. Neue Wege für Ärzte und Patienten Im Gesundheitswesen*.

[5] Edwards A, Elwyn G, Edwards A, Elwyn G (2009). Shared decision-making in health care: achieving evidence-based patient choice. *Shared Decision-Making in Health Care: Achieving Evidence-Based Patient Choice*.

[6] Elwyn GJ, Edwards A, Kinnersley P, Grol R (2000). Shared decision making and the concept of equipoise: the competences of involving patients in healthcare choices. *The British Journal of General Practice*.

[7] Colter A (1999). Paternalism or Partnership? Patients have grown up—and there's no going back. *British Medical Journal*.

[8] Klemperer D, Rosenwirth M (2005). *Shared Decision Making: Konzept, Voraussetzungen und Politische Implikationen*.

[9] Bieber C, Ringel N, Eich W (2007). Shared decision making and the furthering of its implementation in the health service—the patients’ desire, the politics’ request. *Klinikarzt*.

[10] Murray E, Charles C, Gafni A (2006). Shared decision-making in primary care: tailoring the Charles et al. model to fit the context of general practice. *Patient Education and Counseling*.

[11] Simon D, Loh A, Härter M (2008). Development and evaluation of interventions to support shared decision making—framework and measuring instruments. *Zeitschrift für Medizinische Psychologie*.

[12] Matthys J, Elwyn G, van Nuland M (2009). Patients’ ideas, concerns, and expectations (ICE) in general practice: impact on prescribing. *The British Journal of General Practice*.

[13] Bertrakis KD, Azari R (2011). Determinants and outcomes of patient-centered care. *Patient Education and Counseling*.

[14] Butzlaff M, Floer B, Isfort J, Böcken J, Braun B, Schnee M (2003). Shared decision making: der patient im mittelpunkt von gesundheitswesen und praxisalltag. *Gesundheitsmonitor 2003*.

[15] Dierks ML, Seidel G, Härter M, Loh A, Spies C (2005). Gleichberechtigte Beziehungsgestaltung zwischen Ärzten und Patienten—wollen Patienten wirklich Partner sein?. *Gemeinsam Entscheiden—Erfolgreich Behandeln*.

[16] Schneider A, Körner T, Mehring M, Wensing M, Elwyn G, Szecsenyi J (2006). Impact of age, health locus of control and psychological co-morbidity on patients’ preferences for shared decision making in general practice. *Patient Education and Counseling*.

[17] Bastiaens H, van Royen P, Pavlic DR, Raposo V, Baker R (2007). Older people’s preferences for involvement in their own care: a qualitative study in primary health care in 11 European countries. *Patient Education and Counseling*.

[18] Voigt I, Wrede J, Diederichs-Egidi H, Dierks ML, Hummers-Pradier E, Junius-Walker U (2010). PrefCheck: patient-centered treatment planning with older multimorbid patients. *Zeitschrift für Gerontologie und Geriatrie*.

[19] Junius U, Fischer G (2002). Geriatric assessment for use in primary case—results of a concerted action involving seven European countries. *Zeitschrift für Gerontologie und Geriatrie*.

[20] Seidel G, Möller S, Schneider N, Buser K, Walter U, Dierks ML Beteiligung an gesundheitsbezogenen Entscheidungen—die Perspektive hochaltriger Patienten.

[21] Mayring P (2008). *Qualitative Inhaltsanalyse: Grundlagen und Techniken*.

[22] Kluge S (2000). Empirisch begründete Typenbildung in der qualitativen Sozialforschung. *Forum: Qualitative Social Research*.

[23] Kelle U, Kluge S (2010). *Vom Einzelfall zum Typus: Fallvergleich und Fallkontrastierung in der Qualitativen Sozialforschung*.

[24] Weber M (1904). *Die Objektivität Sozialwissenschaftlicher und Sozialpolitischer Erkenntnis*.

[25] Dierks ML (1995). *Frauen und Krebsfrüherkennung—Eine Typologie [Ph.D. thesis]*.

[26] Jöckel KH, Babitsch B, Bellach BM, Bloomfield K, Hoffmeyer-Zlotnik J, Winkler J, Ahrens W, Bellach BM, Jöckel KH (1998). Messungen und Quantifizierungen soziographischer Merkmale in epidemiologischen Studien. *Messung Soziographischer Merkmale in der Epidemiologie*.

[27] Flynn KE, Smith MA, Vanness D (2006). A typology of preferences for participation in healthcare decision making. *Social Science and Medicine*.

[28] Scheibler F (2003). *Shared Decision Making: von der Compliance zur Partnerschaftlichen Entscheidungsfindung*.

[29] Whitney SN, McGuire AL, McCullough LB (2004). A typology of shared decision making, informed consent, and simple consent. *Annals of Internal Medicine*.

[30] Cvengros JA, Christensen AJ, Hillis SL, Rosenthal GE (2007). Patient and physician attitudes in the health care context: attitudinal symmetry predicts patient satisfaction and adherence. *Annals of Behavioral Medicine*.

[31] Marstedt G, Buitkamp M, Braun B, Böcken J, Braun B, Amhof R (2007). Eine patiententypologie: befunde zur differenzierung unterschiedlicher normen und verhaltensmuster von patienten im gesundheitssystem. *Gesundheitsmonitor 2007: Gesundheitsversorgung und Gestaltungsoptionen aus der Perspektive von Bevölkerung und Ärzten*.

[32] Stewart M (2001). Towards a global definition of patient centred care: the patient should be the judge of patient centred care. *British Medical Journal*.

[33] Clayton MF, Latimer S, Dunn TW, Haas L (2011). Assessing patient-centered communication in a family practice setting: how do we measure it, and whose opinion matters?. *Patient Education and Counseling*.

[34] Jagosh J, Donald Boudreau J, Steinert Y, Macdonald ME, Ingram L (2011). The importance of physician listening from the patients' perspective: enhancing diagnosis, healing, and the doctor-patient relationship. *Patient Education and Counseling*.

[35] Shaw A, Ibrahim S, Reid F, Ussher M, Rowlands G (2009). Patients’ perspectives of the doctor-patient relationship and information giving across a range of literacy levels. *Patient Education and Counseling*.

[36] Große K, Rendenbach U (2006). Dem (Haus-)arzt vertrauen. *Notfall & Hausarztmedizin*.

[37] Schwan G (2006). Ohne Vertrauen heilen?. *Das Gesundheitswesen*.

[38] Engelhardt K (2010). Patient-centered medicine: a physician’s challenge. *Deutsche Medizinische Wochenschrift*.

